# Binary effects of intravascular laser irradiation of blood on motor recovery and homocysteine reduction in a case with ischemic hemiparesis: portrayed with brain perfusion images

**DOI:** 10.1186/s12883-022-02896-8

**Published:** 2022-09-27

**Authors:** Sheng-Wen A. Li, Yen-Po Lin, Shih-Po Hsieh, Shin-Tsu Chang

**Affiliations:** 1 School of Medicine, National Defense Medical Center, Taipei, Taiwan; 2 School of Medicine, Chung-Shan Medical University, Taichung, Taiwan; 3 Department of Physical Medicine and Rehabilitation, Kaohsiung Veterans General Hospital, No.386, Dazhong 1st Rd., Zuoying Dist., Kaohsiung, 813414 Taiwan; 4 Department of Physical Medicine and Rehabilitation, Tri-Service General Hospital School of Medicine, National Defense Medical Center, Neihu District, No. 161, Section 6, Minquan East Road, Taipei, 114201 Taiwan

**Keywords:** Intravascular laser irradiation of blood, Homocysteine, Corona radiata, Hemiparetic stroke, Cerebral hemisphere, Single photon emission computed tomography

## Abstract

**Background:**

Stroke is a burdensome cerebral eventthat affects many aspects of daily activities such as motion, speech, memory, vision, and cognition. Intravascular laser irradiation of blood (ILIB) is a novel therapy, going beyond conventional rehabilitation modalities, that is effective in stroke recovery. Homocysteine ​​is an important risk factor associated with stroke. However, there are few studies that examine the relationship between ILIB treatment and the level of homocysteine. In recent years, researchers use the single-photon emission computed tomography (SPECT) scan of the brain to evaluate stroke patients and patients with a neurologicdeficit. The present report investigates the clinical effect of ILIB treatment on the level of serum homocysteine, the perfusion change of impaired brain region via SPECT, and the patient’s neurologic appearance.

**Casepresentation:**

We focus on a case of a 62-year-old man with subacute stroke accompanied with left hemiparesis and hyperhomocysteinemia, who showed dramatic improvement in muscle power, a decreasing level of homocysteine, and increased blood flow of the right cerebral after three-courseILIB treatment.

**Conclusion:**

We found that ILIB is effective in lowering serum levels of homocysteine and facilitating cerebral circulation for the patient with subacute stroke.

## Background

Stroke is a common cerebral event that occurs to millions of people globally, which may provoke partial paralysis, affect speech function, memory, or visual loss.People who have a stroke may be unable to perform daily activities, consequently becoming a burden to family caregivers, which affects their physical, emotional, social, and financial wellbeing [1].Rehabilitation plays an important role in helping patients achieve their best recovery and return to normal daily activities.Physical therapy, occupational therapy, and speech therapy were common rehabilitation disciplines. Complementary and alternative interventions such as intravascular laser irradiation of blood, acupuncture, Chinese herbal medicine, yoga, and Tai-Chi also offer efficacy in improving patient outcomes [2].

Homocysteine is an amino acid derived from the metabolism of dietary methionine, and it is a well-known risk factorassociated withatherothrombotic vascular events. Deleterious effects on vascular endothelial cell function were discovered in both animal models and human studies with a high level of homocysteine [3]. An elevated homocysteine level increases the risk of recurrent ischemic stroke in patients experiencing the convalescent phase of acute stroke [4]. Therefore, it is vital to understand how to decrease the level of homocysteine in clinical practice.

Intravascular laser irradiation of blood (ILIB) is an alternative therapy for various illnesses such as stroke, pain, spinal cord injury, diaschisis [5], sleep disturbance [6], delayed neurological sequelae [7], cognitive dysfunction after brain tumor [8], and Sjögren's syndrome [9]. However, the association between ILIB and the level of homocysteine remains unknown. In this report, we present a case of a patient with subacute stroke accompanied by left hemiparesis and hyperhomocysteinemia, who underwent ILIB treatment.

## Case presentation

The 62-year-old male patient has no underlying disease. He found his left-hand fingers clumsy and had difficulty performing adduction and flexion while washing his hair on July 23, 2021. Symptoms progressed to his left leg, and he could not maneuver his left leg. He was sent to NCKUH Emergency Room (ER), where the ER doctor considered his symptom as an episode of stroke and arranged a brain computed tomography (CT) scan soon. The CT image showed no sign of intracerebral hemorrhage (ICH). The patient’s initial National Institutes of Health Stroke Scale score was 2 points, and, as a result, he did not receive thrombolytic therapy.

After inpatient admission on July 24, the carotid duplex test showed mild to moderate atherosclerosis without significant hemodynamic change. Dual antiplatelet therapy (aspirin and clopidogrel) was given for stroke. Unfortunately, his symptoms worsened on July 25, 2021, and the patient was paralyzed on the left side of his body with left facial palsy and mild dysarthria. Repeated CT was conducted due to progression of left limb weakness and numbness, and still showed no sign of ICH. Instead, magnetic resonance imaging (MRI) of the brain without contrast showed acute infarction in right corona radiata, together with stenosis of left vertebral artery and basilar artery. Titrated hydration with dual anti-platelet was kept. Statin was applied for newly diagnosed dyslipidemia, as well as an antihypertensive drug was given.

The patient was transferred to the Ministry of Health and Welfare Tainan Hospital for Post-Acute Care on August 6, 2021, where he achieved partial recovery of muscle power. The patient called at our outpatient department for advanced recovery on August 26, 2021, where neurologic examination revealed muscle power grade 3 at left upper limb and 5 at left lower limb. The patient could walk slowly without a cane but had a limited range of motion for flexion and extension of the left foot. He was strenuous for him to raise his hand by himself. He was admitted for neurologic and motor recovery. Laboratory data revealed normal values in most items (included C reactive protein 0.1 mg/d, Creatinine 1.16 mg/dl, Mean corpuscular volume 89.4 fl), except homocysteine as 15.97 μmol/L (normal range: 5-15 μmol/L).

**Fig. 1:**
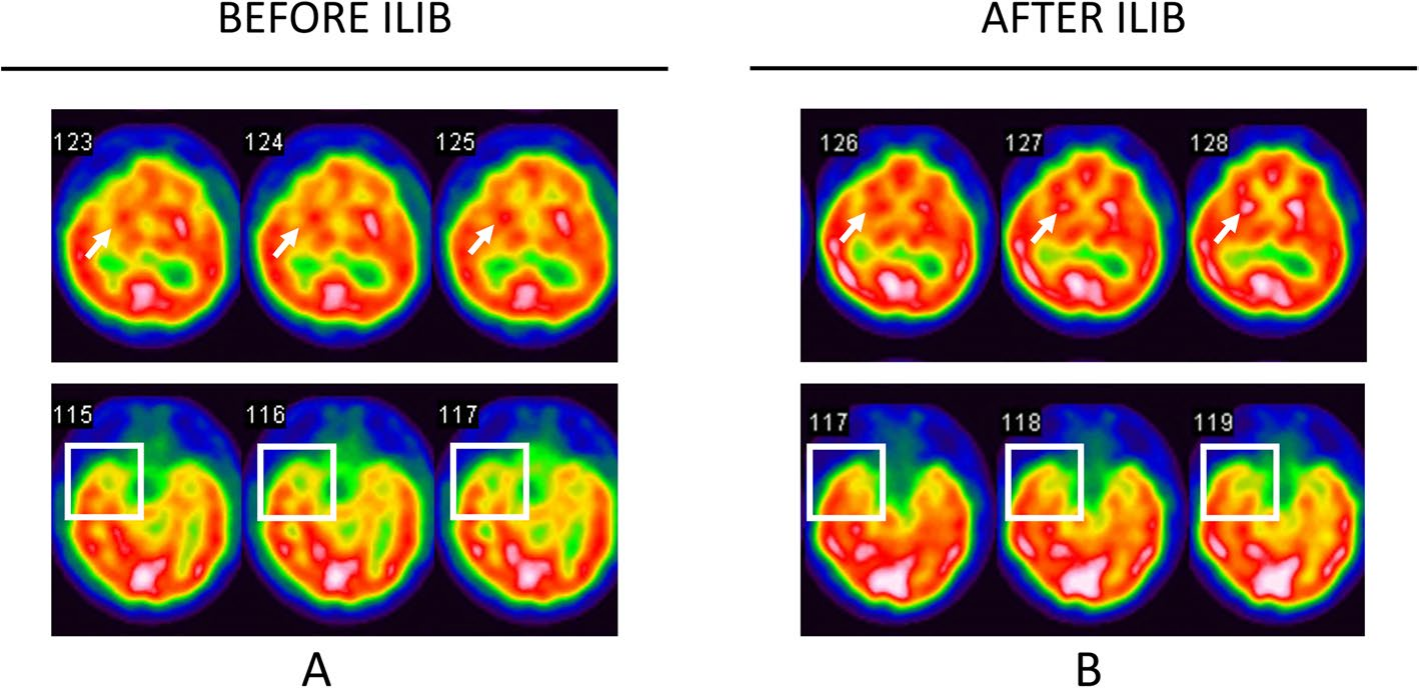
The brain SPECT images of the patient. In **A**, SPECT showed a decrease of the right cerebral blood flow in the right sensorimotor strip, frontal lobe, anterior and posterior cingulate gyri, left medial temporal lobe (white box), corona radiata, basal ganglion (white arrow), and thalamus. In **B**, there is increased uptake in the frontal lobe, temporal lobe, basal ganglion, amygdala, and pons

The brain single-photon emission computed tomography (SPECT) showed a decrease in right cerebral blood flow of right sensorimotor strip, anterior and posterior cingulate gyri, left medial temporal lobe, corona radiata, basal ganglion, and thalamus, as revealed in Fig. 1A.

The comprehensive care plan, including physical therapy, occupational therapy, and language therapy, attained limited improvement of the patient’s condition. Thus, ILIB therapy was added asalternative treatment. After three courses of ILIB therapy, the patient’s condition improved. The following muscle strength was grade 5 over his left arm and grade 4 over his elbow and wrist. Motion range of his lower left feet increased, and muscle power improved to grade 5. The second SPECT showed mild improvement of right cerebral hemisphere blood flow, e.g., frontal lobe, anterior cingulate gyrus, corona radiata, basal ganglion, and thalamus, as shown in Fig. 1B. The patient’s homocysteine level decreased from 15.97 to 10 μmol/L, with stable data result of C reactive protein (0.11 mg/d), Creatinine (1.01 mg/dl), and Mean corpuscular volume (91.0 fl).

## Discussion and conclusions

A comprehensive rehabilitation program is critical to optimize post-stroke outcomes. With the development of new treatment modalities for stroke, ILIB is proposed for cognitive dysfunction, motor dysfunction, and crossed cerebellar diaschisis after acute stroke [5, 10]. However, homocysteine, which has been confirmed as the risk factor of stroke, is not mentioned before and after ILIB treatment in existing literature [11]. To the best of our knowledge, this is the first report using both SPECT and homocysteine to track the efficacy of ILIB in ischemia stroke. Our case is a subacute stroke with left hemiparesis and a high level of homocysteine. Even under physical therapy and occupational therapy, recovery was still limited. To our surprise, after ILIB therapy, the muscle power increased from grade 3 to 5; homocysteine levels decreased from 15.97 to 10 μmol/L, and the following SPECT demonstrate an obvious improvement of right cerebral hemisphere blood flow e.g., anterior cingulate gyrus, basal ganglion, and thalamus.

In the past decades, homocysteine is considered an indicator of vascular disease worldwide [12].Increasing evidence has confirmed that patients with acute stroke and hyperhomocysteinemia are at an increased risk of early neurological deterioration [13]. Considering our case treated with ILIB, the homocysteine level reduced to the normal range. This result was similar to, but not the same with a recently published article reporting a hemorrhagic stroke survivor, which examined serum level of the homocysteine before and after the ILIB and concluded that ILIB might possess a dual effect to increase thalamic perfusion and simultaneously enhance catabolism of homocysteine [14]. In contrast to the aforementioned study regarding ICH, our case is ischemic stroke, which is approximately 87% of stroke, as reported by Grysiewicz et al. [15]. However, medical use of statins, as a secondary stroke prevention, may also cause lower homocysteine concentrations in patients, reported by a latest meta-analysis [16], leading difficulty to clarify the individual effectiveness of ILIB. Despite that further cohort study is required, our result offers an insight into ILIB to reduce serum level of homocysteine in patients with both types of strokes.

Brain SPECT is an effective clinical method, although it is often ignored. The imaging modality has been used in assessing prognosis, in evaluating response to therapy, and in monitoring many neurologic and psychiatric recovery [17]. A prospective study conducted in 1988 first applied SPECT in patients with brainstem stroke and confirmed a significant difference in frontal and parietal hypoperfusion when compared mean counts with age-matched controls [18]. In recent years, researchers use this feasible tool to present the issue of stroke in patients and obtain huge clinical information. In our case, the second SPECT images show remarkable improvement in blood perfusion after the ILIB. Simultaneously, the patient showed a diminish of left hemiparesis and numbness. Moreover, he demonstrated better functional activities in daily life compared with that of the initial evaluation by neurologic examination of muscle power, range of motion and ADL Index.The improvement in our patient symptoms should be correlated with an increase in blood flow of the right frontal lobe, in which the mechanism has been approved by previous study [19]. The injured ischemic motor cortex has a reduced cortical excitability at the acute phase and a suspension of the topographic representation of affected muscles, contributing to worsening of neurological deficit. Thus, when we improve blood perfusion in ischemic stroke patients using ILIB therapy, a great clinical improvement was recorded.

In the present study, the post-treatment imaging study revealed that the ILIB was effective in facilitating circulation. Another case report showsa similar progression in an Impaired blood flow of the basal ganglion after the ILIB [20]. In the case by Sun et al., the patient hasa limited improvement of speech, comprehension, and limbs movement after 1 month of intensive physical, occupational, and speechtherapy. However, after ILIB intervention, the patient's overall functions improved, and SPECT improvedbefore perfusion deficit at the right frontal and parietal regions. Their result may be consistent with ours, indicating that ILIB could be a novel technique to treat patients with ischemic infarction.

Concerning the mechanism of circulation and metabolism of ILIB in an animal study, some new neuronal cells were generated in the subventricular zone ofthe infarcted hemisphere in stroke rats, which received ILIB [21]. Regarding the human study, severalpieces of evidence confirm that ILIB promotes and induces a photobiological response inside cells, activates the production of adenosine triphosphate, and red blood cellsdeformability to pass through tiny blood vessels, and amplifies microcirculation [22]. Although further studies are needed to extend ILIB intreating other diseases, it could be an alternative treatment for patients with stroke.

Our case concerns a subacute stroke with left hemiparesis and a high level of homocysteine. The intervention of ILIB could help the patient to achieve some degree of neurologic recovery and reduce homocysteine, which is considered a risk factor. Brain SPECT plays an important role in appraising recovery. Our case demonstrated a useful method in scintigraphic rehabilitation.

## Data Availability

The datasets used and/or analyzed during the current study are available from the corresponding author on reasonable request.

## Abbreviations

MRI: Magnetic resonance imaging
ICH: Intracerebral hemorrhage
CT: Computed tomography
ER: Emergency Room
ILIB: Intravascular laser irradiation of blood
SPECT: Single-photon emission computed tomography
